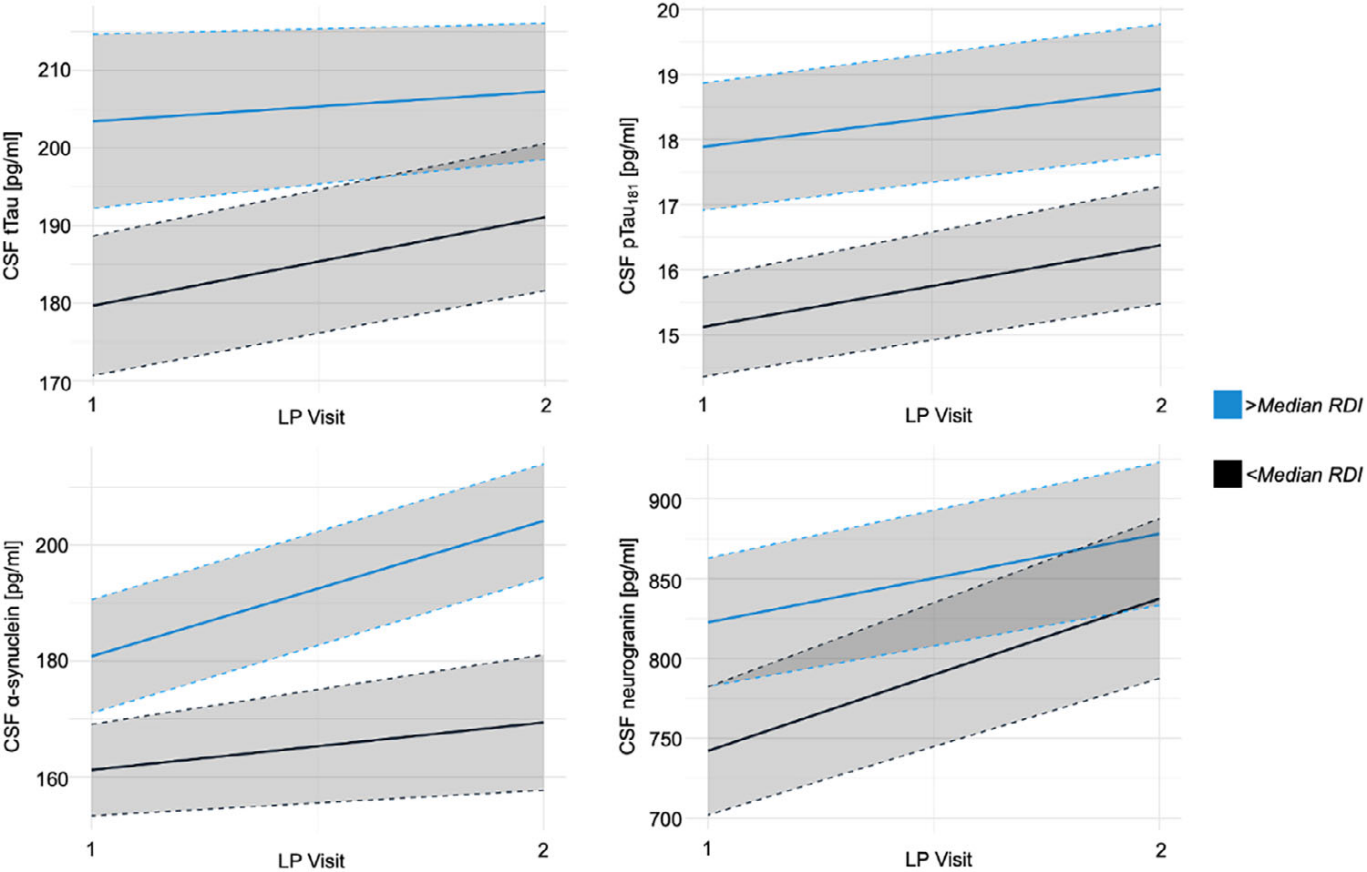# Obstructive sleep apnea severity is associated with CSF biomarkers of synaptic dysfunction and neuroinflammation in a cohort at risk for AD

**DOI:** 10.1002/alz.088835

**Published:** 2025-01-09

**Authors:** Kyle J Edmunds, Brianne M. Breidenbach, Matthew P Glittenberg, Sterling C. Johnson, Sanjay Asthana, Catherine L. Gallagher, Bruce P Hermann, Mark A. Sager, Cynthia M. Carlsson, Gwendlyn Kollmorgen, Clara Quijano‐Rubio, Kaj Blennow, Henrik Zetterberg, Barbara B. Bendlin, Paul E Peppard, Ozioma C Okonkwo

**Affiliations:** ^1^ Wisconsin Alzheimer's Disease Research Center, School of Medicine and Public Health, University of Wisconsin‐Madison, Madison, WI USA; ^2^ Geriatric Research Education and Clinical Center, William S. Middleton Memorial Veterans Hospital, Madison, WI USA; ^3^ Wisconsin Alzheimer’s Institute, University of Wisconsin‐Madison School of Medicine and Public Health, Madison, WI USA; ^4^ Wisconsin Alzheimer's Disease Research Center, Madison, WI USA; ^5^ Geriatric Research Education and Clinical Center William S. Middleton VA Hospital, Madison, WI USA; ^6^ Wisconsin Alzheimer's Disease Research Center, University of Wisconsin School of Medicine and Public Health, Madison, WI USA; ^7^ University of Wisconsin‐Madison, School of Medicine and Public Health, Madison, WI USA; ^8^ Wisconsin Alzheimer's Institute, University of Wisconsin School of Medicine and Public Health, Madison, WI USA; ^9^ Wisconsin Alzheimer’s Institute, University of Wisconsin School of Medicine and Public Health, Madison, WI USA; ^10^ University of Wisconsin‐Madison, Madison, WI USA; ^11^ Roche Diagnostics GmbH, Penzberg Germany; ^12^ Roche Diagnostics International Ltd., Rotkreuz Switzerland; ^13^ Clinical Neurochemistry Laboratory, Sahlgrenska University Hospital, Mölndal Sweden; ^14^ Department of Psychiatry and Neurochemistry, Institute of Neuroscience and Physiology, University of Gothenburg, Mölndal Sweden; ^15^ Institute of Neuroscience and Physiology, University of Gothenburg, Mölndal Sweden; ^16^ Department of Psychiatry and Neurochemistry, Institute of Neuroscience and Physiology, The Sahlgrenska Academy at the University of Gothenburg, Mölndal Sweden; ^17^ Department of Population Health Sciences, School of Medicine and Public Health, University of Wisconsin‐Madison, Madison, WI USA; ^18^ University of Wisconsin, Madison, WI USA

## Abstract

**Background:**

Obstructive sleep apnea (OSA) is associated with hypoxia‐induced neuronal impairment and dysfunction—key risk factors for the pathogeneses of age‐related neurodegenerative diseases such as Alzheimer‘s disease (AD). This study examined longitudinal associations between OSA severity and CSF biomarkers associated with AD, synaptic dysfunction, and neuroinflammation in a sample of late‐middle‐aged adults with increased risk for AD.

**Method:**

N=25 cognitively unimpaired adults (64% female, mean age 65.8 ± 7.1 years, 42.3% APOE‐ε4 carriers) from the Wisconsin Alzheimer‘s Disease Research Center (ADRC) participated in overnight polysomnography, where the number of apneas, hypopneas, and respiratory effort related arousals (RERAs) per hour of sleep were recorded. OSA severity was estimated using the base 10 logarithm of the Respiratory Disturbance Index, or log_10_(RDI+1). Following sleep assessment, CSF samples were acquired via lumbar puncture over two subsequent study visits (mean 5.8 years between visits) and analyzed using the NeuroToolKit (NTK), a panel of robust prototype assays (Roche Diagnostics International Ltd), with measures including core AD biomakers—amyloid beta (Aβ)_42_, Aβ_42_:Aβ_40_, pTau_181_, pTau_181_:Aβ_42_, and tTau—as well as neurogranin, neurofilament light (NfL), α‐synuclein, glial fibrillary acidic protein (GFAP), chitinase‐3‐like protein 1 (YKL‐40), soluble triggering receptor expressed on myeloid cells 2 (sTREM2), neuronal pentraxin II (NPTX2), and synaptosome associated protein 25 (SNAP25). Linear mixed effects models (with random intercept and random slope) were assembled to test longitudinal associations, adjusting for age, sex, and APOE‐ε4 carriage.

**Result:**

AD biomarker models indicated that RDI was associated with increased CSF levels of tTau (β=0.084, SE=0.013, p=0.014) and pTau_181_ (β=0.022, SE=0.003, p=0.023), but neither outcome remained robust after Bonferroni correction for multiple comparisons (adjusted α=0.0039). In contrast, models of synaptic dysfunction and neuroinflammation surpassed Bonferroni correction, showing a significant association between RDI and levels of α‐synuclein (β=0.09, SE=0.013, p=0.002) and neurogranin (β=0.13, SE=0.03, p=0.002).

**Conclusion:**

This work provides new evidence for the deleterious influence of OSA on biomarkers of synaptic dysfunction, neuroinflammation, and AD in a sample of late‐middle age to older adults enriched with risk for AD.